# Impact of stoma revision surgery on quality of life: the STICK-II retrospective cohort study

**DOI:** 10.1007/s10151-026-03347-z

**Published:** 2026-05-26

**Authors:** R. D. A. T. van der Kolk, R. van den Berg, S. van Egmond, P. J. Tanis, E. B. Deerenberg

**Affiliations:** 1 Department of Surgery, Erasmus University Medical Center, Dr. Molenwaterplein 40, 3015 GD Rotterdam, The Netherlands; 2 Department of Surgery, Franciscus Gasthuis and Vlietland, Rotterdam, The Netherlands

**Keywords:** Quality of life, Retrospective cohort study, Stoma-related complications, Reoperation, Surgical stomas, Colostomy

## Abstract

**Introduction:**

Stoma placement is a frequently performed procedure. Short- and long-term stoma-related complications affect up to 80% of patients and are known to reduce quality of life (QoL). Stoma revision surgery may be indicated, but its influences on QoL have not been studied.

**Methods:**

The precursor to this study, the **ST**oma appl**I**an**C**e lea**K**age and its risk factors, consequences and preventive treatments (STICK-I) was a retrospective cohort study conducted across three hospitals between 2019 and 2022. Patients with a colostomy, ileostomy, or certain type of urostomy for at least 1 year were included. The data of the STICK-I study were used for this substudy. The stoma-QoL score was compared between a stoma revision surgery and a control group using propensity matching. Patients stoma-QoL scores before and after revision surgery were separately analyzed as their own controls.

**Results:**

Out of 643 patients, 336 completed the questionnaire, of whom 65 were assigned to the revision group, 216 to the control group, and 10 to the own-control group. Matching resulted in comparable baseline parameters, except for sex. Other baseline characteristics were equally distributed among the groups. The stoma-QoL scores in patients who had undergone stoma revision (mean 56, 95% CI 52–60) did not significantly differ from those who did not undergo stoma revision (mean 59, 95% CI 57–61), (mean difference 2.61, *p* = 0.160). Patients in the own-control group scored significantly higher on the stoma-QoL after revision surgery (mean 62, 95% CI 52–71) compared with before (mean 55, 95% CI 45–65), (mean difference 6.90, *p* = 0.048).

**Conclusions:**

Postoperative stoma-QoL scores were similar to controls who did not undergo revision. Furthermore, stoma-QoL scores significantly improved after stoma revision surgery. Postoperative stoma-QoL scores in the matched cohort did not differ significantly from controls; a small within-patient sample showed a clinically relevant increase after revision. These exploratory findings suggest that QoL improves after revision, but prospective studies with standardized pre- and postoperative assessment are required to confirm this.

**Supplementary Information:**

The online version contains supplementary material available at 10.1007/s10151-026-03347-z.

## Introduction

In the Netherlands the prevalence of stoma formation is approximately 38,000, with an annual incidence of 7000 cases [1]. Indications for stoma formation are diverse and include diseases of the small bowel, colon, rectum, bladder, and gynecologic diseases involving the bowel. Some examples are malignancy, inflammatory diseases, ischemia, trauma, and incontinence [2, 3]. The most frequently created stomas include end colostomy, loop colostomy, end ileostomy, loop ileostomy, and ileal conduit [2, 3]. Although stoma formation is a widely used surgical intervention, it is associated with various complications with a reported incidence up to 80% [4]. Examples of stoma-related complications requiring surgery include necrosis, stoma dehiscence, retraction, prolapse, parastomal hernia, stenosis, and bleeding [5]. In some cases, these complications necessitate revision surgery.

Stoma formation is known to impact patients’ quality of life (QoL) [6–11] and specific stoma-related complications have been associated with a reduction of QoL [12, 13]. A study by Kald et al. [12] showed a statistically significant difference of the stoma-QoL when comparing patients with and without bulging. Similarly, Van Dijk et al. [13] reported a statistically significant difference on all four domains of the physical QoL of the Short Form Health Survey (SF-36) when comparing patients with and without a parastomal hernia. However, it remains unclear whether patients who undergo stoma revision surgery have worse QoL compared with those who did not have revision surgery, and whether their QoL improves after stoma revision.

Therefore, the primary objective of this study was to compare QoL between stoma patients with and without the need for stoma revision surgery and to compare pre- and postoperative QoL in patients undergoing stoma revision surgery. As a secondary objective, we aimed to evaluate whether QoL outcomes depend on the indication for revision surgery.

## Methods

### Patients

The STICK-II study is a substudy of the STICK-I (**ST**oma appl**I**an**C**e lea**K**age and its risk factors, consequences and preventive treatments). The STICK-I study is a retrospective multicenter study that started in 2019 in the Netherlands. The data of the STICK-I study was used for this substudy. Patients were recruited from three hospitals in Rotterdam, the Netherlands: Franciscus Gasthuis & Vlietland hospital, Maasstad hospital, and Erasmus University Medical Center. Eligible participants were adults (≥ 18 years) who had received an end or loop colostomy, an end or loop ileostomy, or an urostomy (Bricker’s urostomy, or cutaneous ureterostomy), with the stoma in place for at least 1 year. Patients who underwent stoma reversal or had esophagostomy were excluded. Eligible patients were identified through hospital medical records and databases, and were invited by telephone to participate in the study between October 2020 and May 2022. Patients who did not answer the phone were invited by mail. The medical ethics committee of the Erasmus University Medical Centre in Rotterdam approved the STICK study; we also obtained approval from the local ethics committees of the participating hospitals. The Strengthening the Reporting of Observational Studies in Epidemiology (STROBE) guidelines [14] for reporting of observational studies were followed.

### Questionnaires

Participants received an invitation letter with an informed consent form and either two or three questionnaires to complete and return. The first questionnaire was the stoma-QoL questionnaire [15] (Appendix 1). Depending on the type of stoma (ileostomy/colostomy and/or urostomy), a second self-designed questionnaire was included (Appendix 2). Both questionnaires were provided in Dutch.

The stoma-QoL questionnaire [15] is a validated questionnaire especially designed for patients with an ileostomy or colostomy. It assesses four domains—sleep, sexual activity, relations to family and close friends, and social relations to other than family and close friends—with a total of 20 questions. Responses are scored on a 4-point Likert scale (always, sometimes, rarely, not at all), with total score ranging from 20 (lowest stoma-QoL) to 80 (highest stoma-QoL).

The stoma-specific questionnaires were designed for the STICK study. These questionnaires included items on baseline patient characteristics at time of completion, whether the stoma placement was planned, whether the stoma site had been marked before surgery, number of stoma leakages annually, bulging of the stoma, retraction of the stoma, prolapse of the stoma, shrinkage of the stoma, and skin problems of the stoma. Additional baseline characteristics were extracted from medical records: gender, age, center, stoma type, stoma indication, operation type, surgical setting, American Society of Anesthesiologists physical status classification (ASA), time living with a stoma upon completion of the questionnaire, time between completion of the stoma-QoL questionnaire before and after revision surgery, time between stoma placement and first revision surgery, number of revision surgeries, first revision surgery indication, body mass index (BMI), smoking status, pack-years, and medical history.

Patients who met the inclusion criteria, signed the informed consent form, and completed the stoma-QoL questionnaire were assigned to the appropriate group (own-control group, revision group or non-revision control group). Group assignment was determined by whether revision surgery had taken place and by the timing of questionnaire completion. Patients who did not fully complete the stoma-QoL questionnaire were excluded from this study.

### Primary outcome and patient selection

The primary objective of this study was to compare QoL between stoma patients with and without the need for stoma revision surgery and to compare pre- and postoperative QoL in patients undergoing stoma revision surgery. The stoma-QoL was calculated from the stoma-QoL questionnaire [15]. Each question answered with “always” was worth 1 point, with “sometimes” 2 points, with “rarely” 3 points, and with “not at all” 4 points. These points were added to form a total score.

**Fig. 1:**
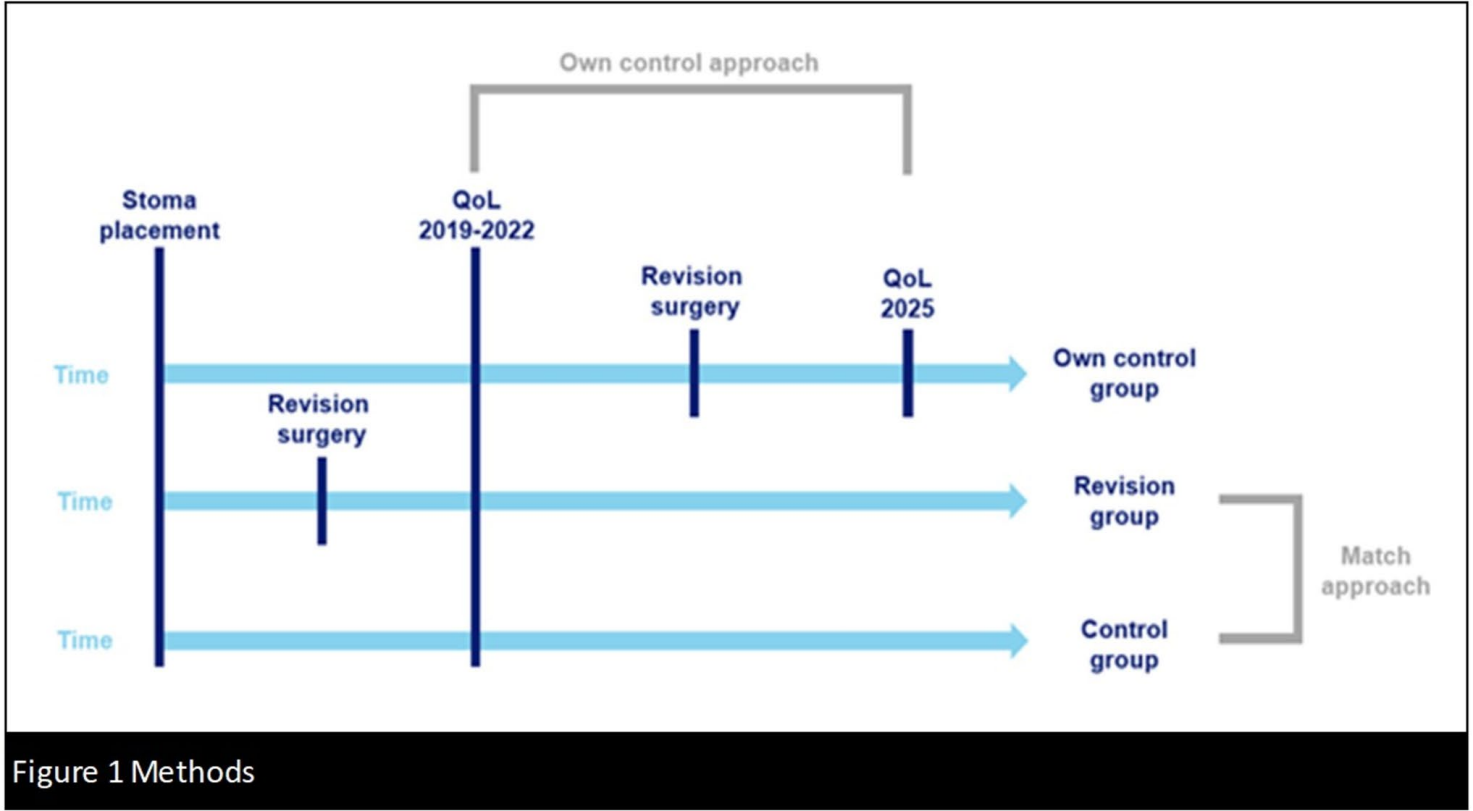
Methods

Patients who underwent a stoma revision surgery any time before the completion of the initial questionnaires were selected and assigned to the revision group. Patients who did not undergo stoma revision surgery were assigned to the control group (Fig. 1). Matching was applied for comparative analysis of these two groups.

To evaluate the impact of revision surgery with the patients serving as their own controls, patients were selected and assigned to the own-control group if they had undergone a stoma revision surgery after completion of the initial questionnaires. Patients were asked to complete the stoma-QoL questionnaire for the second time, but now after undergoing stoma revision.

For subgroup analyses regarding type of revision surgery, patients who underwent parastomal hernia repair in the matched population were selected, as this was the most common indication. Additional subgroup analyses were performed on the basis of the different stoma types in the matched groups. Subgroup analyses could not be performed for patients who served as their own controls due to the small sample size.

### Statistical analysis

Statistical analysis was done using IBM SPSS Statistics version 28.0.1.0 and R-studio version 4.3.2. Numerical variables were reported as the mean and standard deviation (± SD). Categorical variables were reported as frequency (%). For the comparison between results the mean difference (MD) or absolute standardized mean difference (ASMD) was reported. For all analyses, a *p* value of < 0.05 was considered to be statistically significant.

Missing data for categorical variables were imputed using polynomial regression, while continuous variables were imputed using the predictive mean matching method. For each missing piece of data 20 imputations were performed. The following variables were included in the imputation model: age, gender, ASA classification, BMI, diabetes, smoking status, stoma type, stoma indication, duration of stoma existence, number of stoma leakages per year, surgical setting, operation type, and time between the last operation and questionnaire completion.

Matching was performed using propensity score matching. The following variables were included in the propensity score model: age, gender, BMI, stoma type, stoma indication, operation type, and number of stoma leakages per year. The propensity scores of the imputed datasets were collected and patients who underwent stoma revision surgery were matched to controls by the optimal full matching method [16]. With this method, the number of patients discarded from the analysis is minimized and there is no need for 1:k matching. Patients who could not be matched were outliers and were excluded from further analyses. The quality of the matching model was evaluated visually using an absolute standardized mean difference plot. The outcomes were assessed using linear regression.

For the parastomal hernia subgroup, fewer variables were included in the propensity score model due to a smaller sample size. The following variables were included: gender, stoma type, and operation type.

For analysis of the primary endpoint in the group of patients who formed their own controls, the paired *t*-test was used in case of a normal distribution, while the Wilcoxon signed ranks test was used for non-normally distributed data. To decide the cutoff point of clinically relevant change, the minimal clinically important difference (MCID) [17] was determined on the basis of the standard deviation (SD) of the mean change; a value of 0.5 SD was used, as this threshold has been suggested to correspond to the MCID.

## Results

### Baseline characteristics

**Fig. 2:**
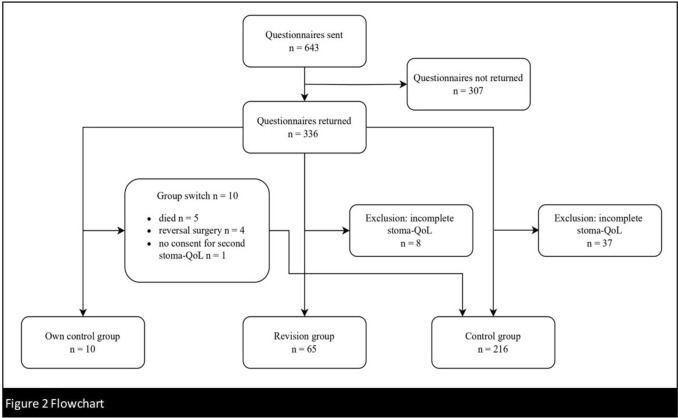
Flowchart

Out of 643 screened patients, 336 (52.3%) provided informed consent and completed and returned the questionnaires (Fig. 2). A total number of 316 patients were potentially eligible for the matched groups. A total of 45 patients were excluded due to an incomplete stoma-QoL questionnaire, after which 65 patients underwent revision surgery and 216 control patients remained available for matching; 10 patients completed a questionnaire both before and after revision surgery and were included for analyses with the patients as their own controls.

The baseline characteristics of the revision and control group before and after matching, as well as the patients who served as their own controls, are presented in Table 1. Before matching, several characteristics were significantly different between the revision and control group. Before matching, patients who had undergone revision surgery were more frequently female (63.1% versus 39.4%), had more often an end ileostomy (21.5% versus 13.0%) or loop ileostomy (18.5% versus 3.7%), and less often an end colostomy (38.5% versus 55.1%) or Bricker urostomy (9.2% versus 25.5%). Furthermore, patients who had undergone revision surgery were less often treated for malignancy (30.2% versus 50.9%), and more often for inflammatory bowel diseases (17.5% versus 8.4%). The proportion of open surgery was similar in both groups (53.1% versus 47.2%), and minimally invasive surgery was more often robot-assisted in the control group (10.2% versus 29.2%). The patients of the revision group had annually more stoma leakages compared to the control group (54 versus 15).

**Table 1:** Baseline characteristics

		Unadjusted				Adjusted		
	Own control^a^	Revision^a^	Control^a^	ASMD^c^	*p*-Value^b^	Revision^a^	Control^a^	ASMD^b^
	*n* = 10	*n* = 65	*n* = 216			*n* = 63	*n* = 214	
Distance				0.886				0.001
Gender					0.001			
Male	4 (40.0)	24 (36.9)	131 (60.6)	0.492		(45.1)	(55.8)	0.222
Female	6 (60.0)	41 (63.1)	85 (39.4)	0.492		(54.9)	(44.2)	0.222
Age	63±13	67±13	70±13	0.234	0.110	67	68	0.010
Center					0.485			
Franciscus Gasthuis & Vlietland	6 (60.0)	35 (53.8)	98 (45.4)					
Erasmus University Medical Center	2 (20.0)	17 (26.2)	68 (31.5)					
Maasstad Hospital	2 (20.0)	13 (20.0)	50 (23.1)					
Stoma type					0.001			
End colostomy	3 (30.0)	25 (38.5)	119 (55.1)	0.342		(47.1)	(49.9)	0.059
End ileostomy	4 (40.0)	14 (21.5)	28 (13.0)	0.209		(21.6)	(15.6)	0.144
Bricker urostomy	1 (10.0)	6 (9.2)	55 (25.5)	0.561		(11.8)	(8.7)	0.107
Loop colostomy		6 (9.2)	4 (1.9)	0.255		(3.9)	(6.2)	0.077
Loop ileostomy	1 (10.0)	12 (18.5)	8 (3.7)	0.380		(15.7)	(19.6)	0.101
Urethrocutaneous stoma			2 (0.9)	0.110				
End colostomy & Bricker	1 (10.0)	1 (1.5)		0.125				
End ileostomy & Bricker		1 (1.5)		0.125				
Stoma indication					0.047			
Malignancy	4 (40.0)	19 (30.2)	109 (50.9)	0.487		(35.3)	(34.9)	0.009
Colorectal	3 (75.0)	14 (73.7)	60 (55.0)					
Urological	1 (25.0)	4 (21.1)	38 (34.9)					
Gynecological		1 (5.3)	7 (6.4)					
Other			4 (3.7)					
Inflammatory bowel disease	4 (40.0)	11 (17.5)	18 (8.4)	0.229		(13.7)	(12.5)	0.032
Ulcerative colitis	2 (50.0)	3 (27.3)	14 (77.8)					
Crohn’s disease	2 (50.0)	8 (72.7)	4 (22.2)					
Inflammation	1 (10.0)	8 (12.7)	28 (13.1)	0.020		(13.7)	(14.0)	0.009
Diverticulitis	1 (100.0)	7 (87.5)	14 (50.0)					
Enterocutaneous fistula		1 (12.5)	10 ( 37.50					
Other			4 (14.3)					
Complication		11 (17.5)	24 (11.2)	0.222		(17.7)	(22.1)	0.111
Anastomotic leak		8 (72.7)	12 (50.0)					
Perforation			5 (20.8)					
Other		3 (27.3)	7 (29.2)					
Excretion problems	1 (10.0)	10 (15.9)	20 (9.3)	0.170		(11.8)	(10.3)	0.042
Fecal incontinence		2 (20.0)	5 (25.0)					
Urinary incontinence	1 (100.0)	2 (20.0)	3 (15.0)					
Constipation		2 (20.0)	2 (10.0)					
Other		4 (40.0)	10 (50.0)					
Obstruction		1 (1.6)	8 (3.7)	0.176		(2.0)	(1.9)	0.005
Other		3 (4.8)	7 (3.3)	0.066		(5.9)	(4.3)	0.075
Operation type					0.021			
Open	3 (30.0)	26 (53.1)	92 (47.2)	0.130		(52.9)	(56.2)	0.065
Laparoscopic	3 (30.0)	10 (20.4)	33 (16.9)	0.058		(21.6)	(20.2)	0.037
Robotic	4 (40.0)	5 (10.2)	57 (29.2)	0.608		(13.7)	(11.9)	0.060
Laparoscopic converted		4 (8.2)	9 (4.6)	0.063		(5.9)	(3.4)	0.104
Robotic converted			1 (0.5)	0.110				
Other		4 (8.2)	3 (1.5)	0.388		(5.9)	(8.4)	0.077
Missing		16	21					
Surgical setting				0.733				
Acute	2 (20.0)	20 (36.4)	65 (30.8)					
Elective	8 (80.0)	34 (61.8)	142 (67.3)					
Semi-elective		1 (1.8)	4 (1.9)					
Missing		10	5					
ASA score				0.985				
I	1 (16.7)	2 (6.9)	8 (5.6)					
II	3 (50.0)	17 (58.6)	88 (62.0)					
III	2 (33.3)	9 (31.0)	41 (28.9)					
IV		1 (3.4)	5 (3.5)					
Missing	4	36	74					
Planned stoma placement	9 (90.0)	52 (80.0)	170 (80.2)		0.928			
Stoma place was marked	9 (90.0)	46 (70.8)	169 (78.2)		0.111			
Number of stoma leaks annually	15±22	54±125	15±46	0.304	0.018	27	20	0.053
Bulging of the stoma	6 (75.0)	31 (52.5)	92 (46.9)		0.522			
Missing	2	6	20					
Retraction of the stoma	2 (22.2)	10 (16.9)	18 (9.3)		0.075			
Missing	1	6	23					
Prolapse of the stoma	5 (55.6)	18 (30.0)	74 (37.6)		0.249			
Missing	1	5	19					
Shrinkage of the stoma	2 (25.0)	7 (12.3)	21 (11.5)		0.835			
Missing	2	8	33					
Skinproblems				0.011				
Never	1 (10.0)	7 (10.9)	45 (20.8)					
Seldom	4 (40.0)	17 (26.6)	55 (25.5)					
Sometimes	4 (40.0)	19 (29.7)	82 (38.0)					
Often	1 (10.0)	10 (15.6)	24 (11.1)					
Very often		11 (17.2)	10 (4.6)					
Time living with a stoma	47±10	99±100	48±30	0.001				
Time between both stoma-QoL's	35±6							
Time between placement and first revision	37±26	20±20						
Number of revision surgeries	1±0	2±1						
First revision surgery indication								
Parastomal hernia	8 (80.0)	17 (27.0)						
Stoma stenosis	1 (10.0)	2 (3.2)						
Stoma retraction	1 (10.0)	1 (1.6)						
Stoma reversal		10 (15.9)						
Stoma necrosis		7 (11.1)						
Anastomotic leak		4 (6.3)						
Stoma prolapse		4 (6.3)						
Ileus		4 (6.3)						
Skin problems		4 (6.3)						
Malignancy		3 (4.8)						
Other		7 (11.1)						
Body mass index	26.9 ± 3.4	26.7 ± 5.2	26.6 ± 5.0	0.128	0.938	26.1	26.7	0.098
Missing		21	44					
Smoking				0.367				
Never	2 (20.0)	19 (30.2)	70 (36.1)					
Quit smoking	5 (50.0)	30 (47.6)	95 (49.0)					
Smoking	3 (30.0)	14 (22.20	29 (14.9)					
Missing		2	22					
Pack-years	16 ± 12	21 ± 19	30 ± 24		0.156			
Missing	3	43	168					
Medical history								
Surgical operation	5 (50.0)	19 (29.2)	43 (20.0)		0.116			
Urological operation		2 (3.1)	12 (5.6)		0.417			
Gynecological operation		8 (12.3)	22 (10.2)		0.636			
Malignancy	1 (10.0)	4 (6.2)	32 (14.8)		0.065			
Gastroenterological		4 (6.2)	21 (9.8)		0.371			
Cardiovascular disease		15 (23.1)	60 (27.9)		0.441			
Diabetes		11 (16.9)	32 (15.0)		0.700			
Pulmonal	1 (10.0)	8 (12.3)	11 (5.1)		0.043			
Dermatological		3 (4.6)	4 (1.9)		0.213			
Neurological		5 (7.7)	17 (7.9)		0.955			
Rheumatological	1 (10.0)	2 (3.1)	6 (2.8)		0.903			
Substance abuse			2 (0.9)		0.435			

a. Data presented as *n* (%) or mean ± SD. Time in months

b. *p*-Values are two-sided. For dichotomous variables, *χ*^2^ test was performed and for continuous variables, independent samples *t*-test

c. *ASMD; absolute* standardized mean difference

**Fig. 3:**
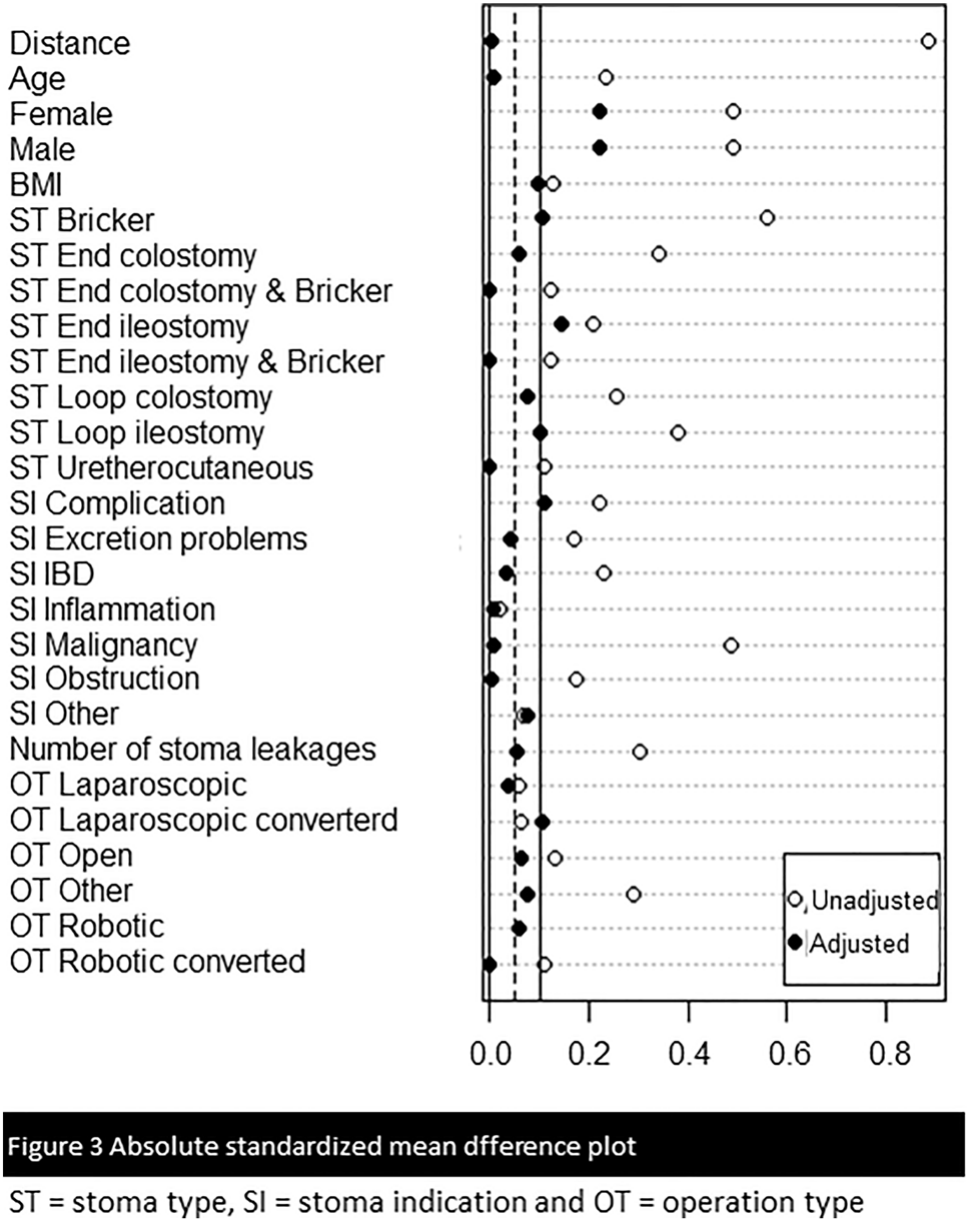
Absolute standardized mean difference plot

Optimal full matching resulted in 63 patients for the adjusted revision group and 214 patients for the adjusted control group. After matching, all baseline characteristics, including number of stoma leakages, were balanced between the groups as the ASMD for the distance equals 0.001 (Table 1, Fig. 3) and the propensity scores were equally distributed (Supplementary 1). After matching, only gender remained significantly different (54.9% versus 44.2% female, ASMD = 0.222) (Table 1).

Out of the ten patients in the own-control group, six were female and the mean age was 63 (SD 13) years (Table 1). Four patients had an end ileostomy, three patients an end colostomy, one patient a Bricker urostomy, one patient a loop ileostomy, and one patient had both an end colostomy and a Bricker urostomy. The indication for the stoma was malignancy in four patients and inflammatory bowel disease in another four patients. For one patient the indication was diverticulitis and for one patient it was urinary incontinence. Most patients received a revision surgery due to parastomal hernia (*n* = 8).

### Stoma quality of life

The stoma-QoL questionnaire was filled out with a median interval from stoma creation of 61 (IQR 43–106) months in the revision group and 42 (IQR 32–54) months in the control group. The time interval between revision surgery and the postoperative stoma-QoL questionnaire was 38 (IQR 24–41) months. Among patients serving as their own controls, the questionnaires were filled out with a median preoperative and postoperative interval relative to revision surgery of 18 and 44 months, respectively.

In the matched population, the stoma-QoL after revision surgery did not significantly differ from the control group (MD 2.6, *p* = 0.160) (Table 2). The power equals 0.999.

**Table 2:** Stoma-QoL revision versus control group

	Mean (SD)	Mean difference (SE)	*p*-Value
Total score Stoma-QoL revision group (*n* = 63)	56.25 (15.35)	2.61 (1.85)	0.160
Total score Stoma-QoL control group (*n* = 214)	58.86 (12.21)		

For the own-control group, patients scored significantly higher on the stoma-QoL after stoma revision surgery compared with before (MD 6.9, 95% CI 0.06–13.74, *p* = 0.048) (Table 3, Supplementary 2). This difference is clinically relevant as the MCID was 4.8. A medium effect size was observed (Cohen’s *d* = 0.72, 95% CI 0.01–1.41), and the statistical power was 0.530.

**Table 3:** Stoma-QoL before versus after stoma revision surgery

	Mean (SD)	Mean difference (95% CI)	*p*-Value
Total score Stoma-QoL before (*n* = 10)	54.80 (14.16)	6.90 (0.06–13.74)	0.048
Total score Stoma-QoL after (*n* = 10)	61.70 (12.89)		

### Parastomal hernia

Subgroup analysis was done for parastomal hernia repair. In the matched population, 17 patients underwent stoma revision surgery due to a parastomal hernia, and 9 of those patients were successfully matched to 93 controls as the ASMD for the distance equals 0.015 (Supplementary 3–5). The stoma-QoL did not significantly differ between the groups. The mean stoma-QoL for the revision group was 52.0 and for the control group 57.2 (MD 5.2, *p* = 0.272) (Supplementary 6). For the own-control approach, the stoma-QoL did not differ significantly before and after stoma revision surgery for this subgroup. The mean stoma-QoL before and after surgery were 56.0 and 62.9, respectively (MD 6.9, 95% CI −0.4 to 14.2, *p* = 0.061).

### Stoma type

For the matched population, the stoma-QoL did not differ significantly between the revision and control group for all but one stoma type (Supplementary 7). Only patients with a Bricker urostomy who had undergone a stoma revision surgery scored significantly higher on the stoma-QoL compared with patients with a Bricker urostomy who had not undergone a stoma revision surgery. The mean stoma-QoL for the patients with a Bricker urostomy who had undergone a stoma revision surgery was 71.2 and the mean stoma-QoL for the patients with a Bricker urostomy who had not undergone a stoma revision surgery was 61.5 (MD 9.7, *p* = 0.047).

## Discussion

The present study evaluated whether stoma revision surgery improves QoL. To our knowledge, this specific question has not been addressed previously. The results of this study indicate that stoma revision surgery may enhance QoL.

This conclusion is supported by the results from the own-control analysis, which suggests a significant increase in stoma-QoL following revision surgery compared with the preoperative questionnaires. Furthermore, these results exceeded the threshold for MCID, which suggests a clinically relevant change. For the matched population, no difference in stoma-QoL was seen between the postoperative scores after revision and the scores in the control group. This might indicate that the stoma-QoL after revision surgery is comparable to the stoma-QoL in the control group. On the basis of our matched approach, we cannot conclude that stoma-QoL increases after revision surgery, it suggests noninferiority. However, this is in accordance with earlier studies [12, 13]. These two studies show that the QoL of patients with stoma complications is lower in comparison with patients without stoma complications. Therefore, when QoL improves for patients with stoma complications—possibly due to revision surgery—the difference in QoL between the two patient groups might vanish.

Moreover, a significant difference in stoma-QoL before and after revision surgery for the subgroup of patients with a parastomal hernia was found, with a* p*-value of 0.061. Additionally, there was no difference in stoma-QoL between the revision and control group for most stoma types. Only the stoma-QoL for patients with a Bricker urostomy and revision surgery was significantly higher compared with patients with a Bricker urostomy and no revision surgery. The underlying cause of this difference remains unclear. A hypothesis is that stoma leakages are more frequent in the Bricker control group compared with the Bricker revision group. However, whether the number of stoma leakages are related to QoL still requires further research.

This study differs from the previous two studies in several aspects [12, 13]. It included a greater variety of stoma types and stoma indications, whereas the study by Kald et al. [12] only included end colostomies, and the study by van Dijk et al. [13] only included end colostomies and patients who underwent a Hartmann procedure or abdominoperineal resection. Furthermore, this is a multicenter study with three centers, while Kald et al. [12] performed a single-center study and van Dijk et al. [13] a multicenter study with two centers. Moreover, cases were matched to controls in the present study to reduce confounding, while this was not incorporated into the other study designs [12, 13].

A limitation of this study is the low power of the own-control analysis, which equals 0.530 due to a low sample size. This power in combination with a relatively high effect size of 0.722 indicates that the chance of a type 1 error, and therefore a false positive result, is higher than ideal. Furthermore, the power is below the threshold for reliable inference. Nevertheless, the power of the match analysis equals 0.999. This high statistical power indicates the analysis was well powered to detect a difference in stoma-QoL before and after revision surgery, if present. As mentioned earlier, the stoma-QoL did not differ between the revision and control group, which suggests that the revision group gained QoL. Given these considerations, the true difference in stoma-QoL is expected to be lower than 6.90 points, but will still be expected to be higher than the MCID of 4.8. Therefore, the results suggest a clinically relevant change.

Despite matching, gender remains statistically imbalanced (ASMD = 0.22). This limitation should be acknowledged and may warrant adjustment in future regression analyses. However, due to the study’s limited statistical power, we were unable to include confounders in the regression model and thus chose not to adjust for this imbalance.

Subgroup analysis for patients with parastomal hernia indicated an improvement in QoL, although statistical significance was not reached. This could be explained due to a small sample size (*n* = 8), which resulted in limited statistical power. Given that the *p*-value of 0.061 is close to the statistical significance threshold, it is likely that a larger sample size would yield statistically significant results. The retrospective design of this study presents additional limitations. For instance, the follow-up time differed, whereas a standardized follow-up period would have minimized potential confounding. Additionally, patients who died during this study could not complete the stoma-QoL for a second time, reducing the sample size and consequently, the statistical power.

Furthermore, call-response bias might have occurred if only patients suffering from stoma leakages had responded to the questionnaires. Considering the use of data of the retrospective multicenter study that started in 2019 in the Netherlands—whose focus is on risk factors for and preventive treatments of stoma leakages—this bias could have led to an overestimation of the observed effects.

Moreover, it is unclear whether patients who underwent revision were symptomatic at baseline. If so, this creates a selection bias favoring post-revision improvement.

In addition, causal inferences cannot be drawn from this observational study. Moreover, retrospective studies have a high risk of recall bias in general. However, this study has no such risk, as the stoma-QoL questionnaire evaluates how patients feel at the time of completing the questionnaire. This constitutes an additional strength of the study.

## Conclusions

To the best of our knowledge, this is the first study that evaluated the influence of stoma revision surgery on QoL in patients with a stoma. Although in a small group of patients the stoma-QoL appeared to improve following revision surgery, the finding that postoperative stoma-QoL scores were comparable to those of the control group that had not undergone revision surgery supports this observation. Future research should seek to confirm these findings through prospective studies with standardized pre- and postoperative QoL assessment to enhance statistical power.

## Supplementary Information

Below is the link to the electronic supplementary material.

Supplementary file1 (DOCX 31 KB)

Supplementary file2 (DOCX 32 KB)

Supplementary file3 (XLSX 103 KB)

## Data Availability

Data used in this study are available upon request.
