# Supplementary material for: Impact of stoma revision surgery on quality of life: the STICK-II retrospective cohort study
Source: Tech Coloproctol. 2026 May 26;30(1):121. doi: 10.1007/s10151-026-03347-z (PMC13388516; doi:10.1007/s10151-026-03347-z)
Supplement: Supplementary file 1 — Supplementary file1 (DOCX 31 KB) [file 10151_2026_3347_MOESM1_ESM.docx]

Gebruiksaanwijzing voor een enquête over de kwaliteit van leven van mensen met een stoma: Stoma – Quality of Life

De enquête ‘Stoma – Quality of Life’ (Kwaliteit van Leven met een stoma) is ontwikkeld om de kwaliteit van leven van mensen met een stoma te meten. De vragen van de enquête zijn het resultaat van een groot aantal interviews met mensen met een stoma. Deze interviews werden gehouden in diverse landen om de meest voorkomende problemen met betrekking tot de kwaliteit van leven voor deze groep mensen aan de orde stellen.

De volgende punten worden behandeld: bezorgdheid over slapen, bezorgdheid over intieme relaties, bezorgdheid over relaties met familie en naaste vrienden en bezorgdheid over relaties met andere mensen dan familie en naaste vrienden.

De vragenlijst bestaat uit 20 vragen. Een voorbeeld van een vraag kan zijn: "Ik ben bang dat het zakje zal loslaten." Alle vragen moeten worden beantwoord op basis van een schaal van 4 punten. De beschikbare opties voor het beantwoorden van elke vraag zijn:

1. Altijd
2. Soms
3. Zelden
4. Helmaal niet

Denk eraan dat **ALLE** 20 vragen moeten worden beantwoord om de vragenlijst bruikbaar te maken. Daarom mogen er geen vragen onbeantwoord blijven. Bovendien mag voor elke vraag **SLECHTS ÉÉN** antwoord worden gegeven.

De vragen zijn zeer eenvoudig en het kost ongeveer 5-10 minuten om de vragenlijst in te vullen.

*Bedankt voor het invullen van de "Stoma- Quality of Life"*

Initialen gebruiker:

Leven met een stoma Enquête over de kwaliteit van leven

voor mensen met ostomie

Datum: / 20


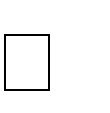

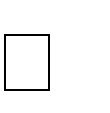

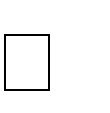

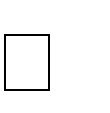

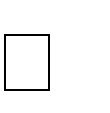

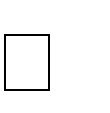

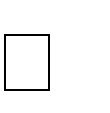

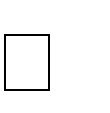

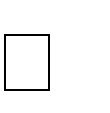

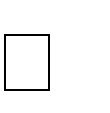

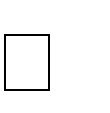

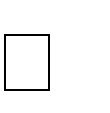

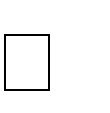

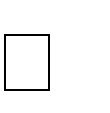

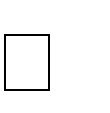

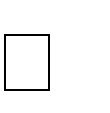

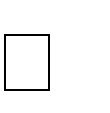

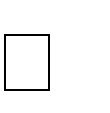

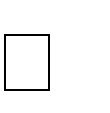

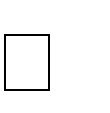

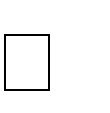

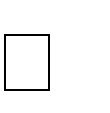

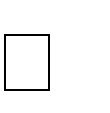

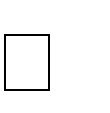

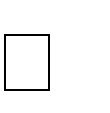

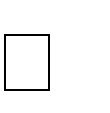

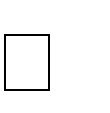

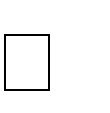

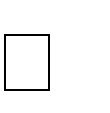

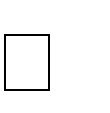

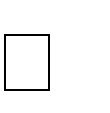

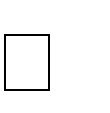

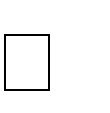

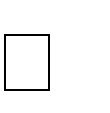

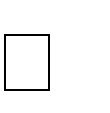

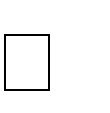

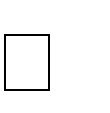

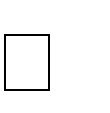

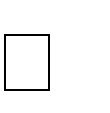

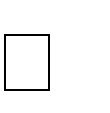
*Kruis het antwoord aan dat het best beschrijft hoe u zich* ***op dit moment*** *voelt*

|  | Altijd | Soms | Zelden | Nooit |
| --- | --- | --- | --- | --- |
| 1. Ik maak me zorgen wanneer het zakje vol is | 1 | 2 | 3 | 4 |
| 2. Ik ben bang dat het zakje zal loslaten | 1 | 2 | 3 | 4 |
| 3. Ik wil altijd weten waar het dichtstbijzijnde toilet is | 1 | 2 | 3 | 4 |
| 4. Ik ben bang dat het zakje een geur zal verspreiden | 1 | 2 | 3 | 4 |
| 5. Ik maak me zorgen om geluiden die afkomstig zijn van de stoma | 1 | 2 | 3 | 4 |
| 6. Ik moet overdag rusten | 1 | 2 | 3 | 4 |
| 7. Mijn stoma beperkt me in de keuze van mijn kleding | 1 | 2 | 3 | 4 |
| 8. Ik voel me overdag moe | 1 | 2 | 3 | 4 |
| 9. Ik voel me seksueel onaantrekkelijk door mijn stoma | 1 | 2 | 3 | 4 |
| 10. Ik kan 's nachts slecht slapen | 1 | 2 | 3 | 4 |
| 11. Ik ben bang dat het zakje ritselt | 1 | 2 | 3 | 4 |
| 12. Ik geneer me voor mijn lichaam vanwege mijn stoma | 1 | 2 | 3 | 4 |
| 13. Ik zou het moeilijk vinden om een nacht van huis te zijn | 1 | 2 | 3 | 4 |
| 14. Het is moeilijk te verbergen dat ik een zakje draag | 1 | 2 | 3 | 4 |
| 15. Ik ben bang dat mijn conditie een belasting is voor de mensen om mij heen | 1 | 2 | 3 | 4 |
| 16. Ik vermijd nauw fysiek contact met mijn vrienden | 1 | 2 | 3 | 4 |
| 17. Mijn stoma maakt het moeilijk voor mij om met andere mensen om te gaan | 1 | 2 | 3 | 4 |
| 18. Ik ben bang om nieuwe mensen te ontmoeten | 1 | 2 | 3 | 4 |
| 19. Ik voel me eenzaam, ook wanneer ik met andere mensen ben | 1 | 2 | 3 | 4 |
| 20. Ik ben bang dat mijn familie zich ongemakkelijk voelt in mijn omgeving | 1 | 2 | 3 | 4 |


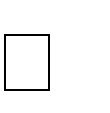

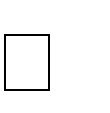

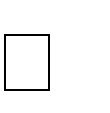

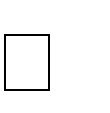

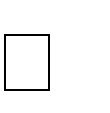

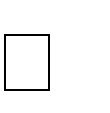

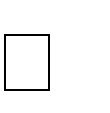

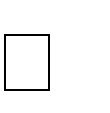

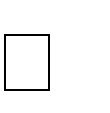

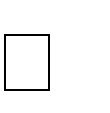

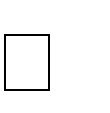

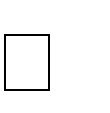

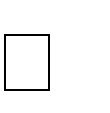

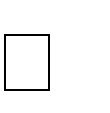

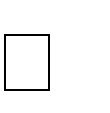

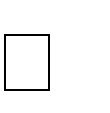

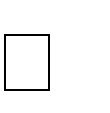

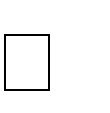

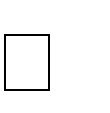

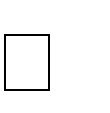

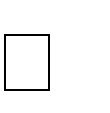

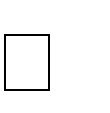

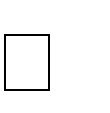

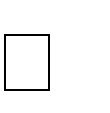

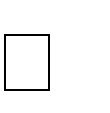

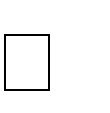

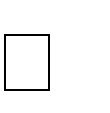

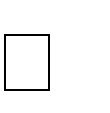

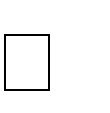

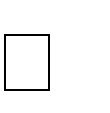

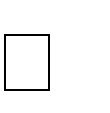

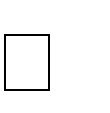

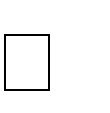

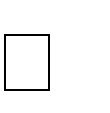

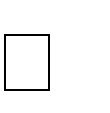

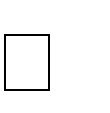

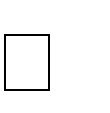

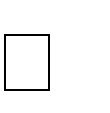

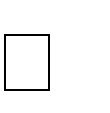

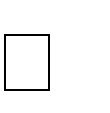
*Beantwoord alle vragen a.u.b. Hartelijk dank voor het invullen van deze enquête.*
