# Supplementary material for: Impact of stoma revision surgery on quality of life: the STICK-II retrospective cohort study
Source: Tech Coloproctol. 2026 May 26;30(1):121. doi: 10.1007/s10151-026-03347-z (PMC13388516; doi:10.1007/s10151-026-03347-z)
Supplement: Supplementary file 2 — Supplementary file2 (DOCX 32 KB) [file 10151_2026_3347_MOESM2_ESM.docx]

**STICK studie vragenlijst colo- en ileostoma**

Versie 1.2, datum: Januari 2020

Naam patiënt: ........................................................................................................................

Geboortedatum: ……………………………………………………………………………………………………………………..

Ziekenhuis: ……………………………………………………………………………………………………………………..

Door middel van onderstaande vragenlijst, de enquête ‘Stoma-Quality of Life’ en de gegevens over uw stoma uit uw patiëntendossier willen wij onderzoeken wat de risicofactoren op het ontstaan van lekkage van ontlasting tussen de stomazak en de huid zijn.

De vragen gaan voornamelijk over uw stoma. De vragenlijst bestaat uit 3 delen (A, B en C), beantwoord a.u.b. elke vraag. Wanneer u twijfelt over het antwoord op een vraag, probeer dan het antwoord te geven dat het meest van toepassing is. Alvast bedankt voor uw medewerking.

***Deel A***

**1. Hoe vaak komt het voor dat de stomazak loslaat van de huid en er lekkage van ontlasting is?**

- Nooit
- Jaarlijks
- Maandelijks
- Wekelijks
- Dagelijks

**2. Kunt u aangeven hoe vaak dit ongeveer per jaar is?**

Aantal keer lekkage van ontlasting per jaar: … keer per jaar

**3. Als u stomazaklekkages hebt, hoezeer heeft dat invloed op de kwaliteit van leven?**

- Helemaal niet van invloed
- Niet erg van invloed
- Redelijk van invloed
- Zeer van invloed
- Uitermate van invloed

**4. Is de frequentie van het loslaten van de stomazak het afgelopen jaar toegenomen, afgenomen of gelijk gebleven?**

- Toegenomen
- Afgenomen
- Gelijk gebleven
- Ik weet het niet

**5. Heeft u moeite met het aanbrengen van de stomazak?**

- Nooit
- Zelden
- Soms
- Vaak
- Zeer vaak

**6. Hoe vaak leegt u gemiddeld de stomazak per dag?**

- 1 keer per dag
- 2 keer per dag
- 3 keer per dag
- 4 keer per dag
- 5 keer per dag
- 6 keer per dag
- 7-8 keer per dag
- 9-10 keer per dag
- Meer dan 10 keer per dag

**7. Hoe vaak vervangt u gemiddeld de stomazak per dag?**

- 1 keer per dag
- 2 keer per dag
- 3 keer per dag
- 4 keer per dag
- 5 keer per dag
- 6 keer per dag
- 7-8 keer per dag
- 9-10 keer per dag
- Meer dan 10 keer per dag

**8. Wat is de productie van uw stoma ongeveer per dag?** Dit kunt u berekenen door het aantal keren legen te vermenigvuldigen met 150 ml. Dit is ongeveer de gemiddelde hoeveelheid als u uw stomazak leegt bij 1/3 vulling. U kunt ook uw stomazak leegmaken boven een maatbeker.

- Tussen 0 en 250 ml per dag
- Tussen 250 en 500 ml per dag
- Tussen 500 en 750 ml per dag
- Tussen 750 en 1000 ml per dag
- Meer dan 1000 ml per dag

**9. Gebruikt u laxerende medicijnen?**

- Ja
- Nee

Zo ja welke? Naam laxerend medicijn: ...............................................................................

**10. Gebruikt u anti-laxerende medicijnen?**

- Ja
- Nee

Zo ja welke? Naam anti-laxerend medicijn: ...............................................................................

***Deel B***

**1. Wat is uw lengte?**

…………… cm

**2. Wat is uw gewicht?**

…………… kg

**3. Kunt u zelf het stoma zien tijdens het verzorgen van de stoma?**

- Ja
- Nee

**4. Kunt u de stomazak zelfstandig legen?**

- Ja
- Nee

**5. Kunt u de stomasysteem zelfstandig wisselen?**

- Ja
- Nee

**6. Voelt u (soms) een bult of uitstulping ter plaatse van of naast het stoma?**

- Ja
- Nee
- Ik weet het niet

**7. Gebruikt u een stoma steunband of steunbroek?**

- Ja
- Nee
- Ik weet het niet

**8. Heeft u huidproblemen rondom de stoma?**

- Nooit
- Zelden
- Soms
- Vaak
- Zeer vaak
- Ik weet het niet

**9. Gebruikt u huidbeschermers zoals crèmes, poeder, sprays of tissues?**

- Ja
- Nee, ga verder naar vraag 11
- Ik weet het niet

**10. Welk type huidbeschermer gebruikt u? (alleen indien *ja* geantwoord bij vorige vraag)**

Naam/merk huidbeschermer: ……………………………………………………………………………………………..

Indien meerdere huidbeschermers: ……………………………………………………………………………………………..

……………………………………………………………………………………………..

……………………………………………………………………………………………..

**11. Hebt u gemerkt dat het stoma ‘gezonken’ is onder het niveau van de huid sinds u ontslagen bent uit het ziekenhuis?**

- Ja
- Nee
- Ik weet het niet

**12. Hebt u gemerkt dat het stoma meer is gaan uitstulpen sinds u ontslagen bent uit het ziekenhuis?**

- Ja
- Nee
- Ik weet het niet

**13. Hebt u gemerkt dat de stoma opening kleiner/nauwer is geworden sinds u ontslagen bent uit het ziekenhuis?**

- Ja
- Nee
- Ik weet het niet

***Deel C***

**1. Bent u voor uw stoma in een ander ziekenhuis onder behandeling dan het ziekenhuis waar de operatie voor het stoma is uitgevoerd?**

- Ja
- Nee

**2. Hoe vaak bezoekt u een stomaverpleegkundige?**

- Nooit
- Jaarlijks
- Eens in de 3 maanden
- Maandelijks
- Wekelijks

**3. Wist u voor de operatie dat u mogelijk een stoma zou krijgen?**

- Ja
- Nee
- Ik weet het niet

**4. Is er voor de operatie een stip getekend op uw buik waar de stoma zou komen?**

- Ja
- Nee
- Ik weet het niet

**5. Heeft u instructies gekregen hoe om te gaan met een stoma?**

- Nooit
- Ja, alleen voor de operatie
- Ja, alleen na de operatie
- Ja, voor én na de operatie

**6. Welke merk opvangmateriaal gebruikt u?**

Naam stomazak: ………..…………………………………………………………………………………………………...

**7. Bestaat dit type opvangsysteem uit een eendelig of tweedelig systeem?**

- Eendelig systeem
- Tweedelig systeem
- Ik weet het niet

**8. Hoe vaak hebt u het type opvangsysteem moeten wisselen sinds u uw stoma heeft?**

- Nooit
- 1 keer
- 2 keer
- 3 keer
- 4 keer
- 5 keer
- Meer dan 5 keer
- Ik weet het niet

**9. Heeft u advies gekregen over beperkingen in werkzaamheden?**

- Nee
- Ja, van de chirurg
- Ja, van de stomaverpleegkundige
- Ja, van de huisarts
- Ja, van de bedrijfsarts
- Ik weet het niet

**10. Zijn er veranderingen in werkzaamheden sinds u een stoma heeft?**

- Nee
- Ja, ik ben minder gaan werken
- Ja, ik ben minder zwaar werk gaan doen
- Ja, ik ben gestopt met werken
- Niet van toepassing

**11. Wat voor werk doet u?** …………………………………………………………………………………………………………

**12. Heeft het hebben van een stoma financiële gevolgen voor u?**

- Nee
- Ja
- Ik weet het niet

**13. Indien u vraag 12 met ‘*Ja’* heeft beantwoord, kunt u dit hieronder verklaren?**

…………………………………………………………………………………………………………………………………………….........

…………………………………………………………………………………………………………………………………………….........

**STICK studie vragenlijst urinestoma**

Versie 1.2, datum: Januari 2020

Naam patiënt: ........................................................................................................................

Geboortedatum: ……………………………………………………………………………………………………………………..

Ziekenhuis: ……………………………………………………………………………………………………………………..

Door middel van onderstaande vragenlijst, de enquête ‘Stoma-Quality of Life’ en de gegevens over uw stoma uit uw patiëntendossier willen wij onderzoeken wat de risicofactoren op het ontstaan van lekkage van urine tussen de stomazak en de huid zijn.

De vragen gaan voornamelijk over uw stoma. De vragenlijst bestaat uit 3 delen (A, B en C), beantwoord a.u.b. elke vraag. Wanneer u twijfelt over het antwoord op een vraag, probeer dan het antwoord te geven dat het meest van toepassing is. Alvast bedankt voor uw medewerking.

***Deel A***

**1. Hoe vaak komt het voor dat de stomazak loslaat van de huid en er lekkage van urine is?**

- Nooit
- Jaarlijks
- Maandelijks
- Wekelijks
- Dagelijks

**2. Kunt u aangeven hoe vaak dit ongeveer per jaar is?**

Aantal keer lekkage van urine per jaar: … keer per jaar

**3. Als u stomazaklekkages hebt, hoezeer heeft dat invloed op de kwaliteit van leven?**

- Helemaal niet van invloed
- Niet erg van invloed
- Redelijk van invloed
- Zeer van invloed
- Uitermate van invloed

**4. Is de frequentie van het loslaten van de stomazak het afgelopen jaar toegenomen, afgenomen of gelijk gebleven?**

- Toegenomen
- Afgenomen
- Gelijk gebleven
- Ik weet het niet

**5. Heeft u moeite met het aanbrengen van de stomazak?**

- Nooit
- Zelden
- Soms
- Vaak
- Zeer vaak

**6. Hoe vaak leegt u gemiddeld de stomazak per dag?**

- 1 keer per dag
- 2 keer per dag
- 3 keer per dag
- 4 keer per dag
- 5 keer per dag
- 6 keer per dag
- 7-8 keer per dag
- 9-10 keer per dag
- Meer dan 10 keer per dag

**7. Hoe vaak vervangt u gemiddeld de stomazak per dag?**

- 1 keer per dag
- 2 keer per dag
- 3 keer per dag
- 4 keer per dag
- 5 keer per dag
- 6 keer per dag
- 7-8 keer per dag
- 9-10 keer per dag
- Meer dan 10 keer per dag

**8. Wat is de productie van uw stoma ongeveer per dag?**

- Tussen 500 ml en 1 liter per dag
- Tussen 1 en 2 liter per dag
- Tussen 2 en 3 liter per dag
- Meer dan 3 liter per dag

***Deel B***

**1. Wat is uw lengte?**

…………… cm

**2. Wat is uw gewicht?**

…………… kg

**3. Kunt u het stoma zien?**

- Ja
- Nee

**4. Kunt u de stomazak zelfstandig legen?**

- Ja
- Nee

**5. Kunt u de stomasysteem zelfstandig wisselen?**

- Ja
- Nee

**6. Voelt u (soms) een bult of uitstulping ter plaatse van of naast het stoma?**

- Ja
- Nee
- Ik weet het niet

**7. Gebruikt u een stoma steunband of steunbroek?**

- Ja
- Nee
- Ik weet het niet

**8. Heeft u huidproblemen rondom het stoma?**

- Nooit
- Zelden
- Soms
- Vaak
- Zeer vaak
- Ik weet het niet

**9. Gebruikt u huidbeschermers zoals crèmes, poeder, sprays of tissues?**

- Ja
- Nee, ga verder naar vraag 11
- Ik weet het niet

**10. Welk type huidbeschermer(s) gebruikt u? (alleen indien *ja* geantwoord bij vorige vraag)**

Naam/merk huidbeschermer: ……………………………………………………………………………………………..

Indien meerdere huidbeschermers: ……………………………………………………………………………………………..

……………………………………………………………………………………………..

……………………………………………………………………………………………..

**11. Hebt u gemerkt dat het stoma ‘gezonken’ is onder het niveau van de huid sinds u ontslagen bent uit het ziekenhuis?**

- Ja
- Nee
- Ik weet het niet

**12. Hebt u gemerkt dat het stoma meer is gaan uitstulpen sinds u ontslagen bent uit het ziekenhuis?**

- Ja
- Nee
- Ik weet het niet

**13. Hebt u gemerkt dat de stoma opening kleiner/nauwer is geworden sinds u ontslagen bent uit het ziekenhuis?**

- Ja
- Nee
- Ik weet het niet

***Deel C***

**1. Bent u voor uw stoma in een ander ziekenhuis onder behandeling dan het ziekenhuis waar de operatie voor het stoma is uitgevoerd?**

- Ja
- Nee

**2. Hoe vaak bezoekt u een stomaverpleegkundige?**

- Nooit
- Jaarlijks
- Eens in de 3 maanden
- Maandelijks
- Wekelijks

**3. Wist u voor de operatie dat u mogelijk een stoma zou krijgen?**

- Ja
- Nee
- Ik weet het niet

**4. Is er voor de operatie een stip getekend op uw buik waar de stoma zou komen?**

- Ja
- Nee
- Ik weet het niet

**5. Heeft u instructies gekregen hoe om te gaan met een stoma?**

- Nooit
- Ja, alleen voor de operatie
- Ja, alleen na de operatie
- Ja, voor én na de operatie

**6. Welke merk opvangmateriaal gebruikt u?**

Naam stomazak: ………..…………………………………………………………………………………………………...

**7. Bestaat dit type opvangsysteem uit een eendelig of tweedelig systeem?**

- Eendelig systeem
- Tweedelig systeem
- Ik weet het niet

**8. Hoe vaak hebt u het type opvangsysteem moeten wisselen sinds u uw stoma heeft?**

- Nooit
- 1 keer
- 2 keer
- 3 keer
- 4 keer
- 5 keer
- Meer dan 5 keer
- Ik weet het niet

**9. Heeft u advies gekregen over beperkingen in werkzaamheden?**

- Nee
- Ja, van de chirurg
- Ja, van de stomaverpleegkundige
- Ja, van de bedrijfsarts
- Ik weet het niet

**10. Zijn er veranderingen in werkzaamheden sinds u een stoma heeft?**

- Nee
- Ja, ik ben minder gaan werken
- Ja, ik ben minder zwaar werk gaan doen
- Ja, ik ben gestopt met werken
- Niet van toepassing

**11. Wat voor werk doet u?** …………………………………………………………………………………………………………

**12. Heeft het hebben van een stoma financiële gevolgen voor u?**

- Nee
- Ja
- Ik weet het niet

**13. Indien u vraag 12 met ‘*Ja’* heeft beantwoord, kunt u dit hieronder verklaren?**

…………………………………………………………………………………………………………………………………………….........

…………………………………………………………………………………………………………………………………………….........
